# Editorials

**Published:** 1890-10

**Authors:** 


### HOMŒOPATHIC PHYSICIAN,

A MONTHLY JOURNAL OF

HOMŒOPATHIC MATERIA MEDICA AND CLINICAL MEDICINE.

"If our school ever gives up the strict inductive method of Hahnemann, we are lost, and deserve only to be mentioned as a caricature in the history of medicine."—CONSTANTINE HERING.

Vol. X. OCTOBER, 1890. No. 10.

### EDITORIALS.

HEREDITY OF TUBERCULOSIS.—At page 53 of this current volume, in connection with remarks respecting the microbic origin of disease, we asked this question: "How often have you seen tuberculosis where there was no history of heredity?"

Dr. August Haupt, of Soden, a German watering place, in a pamphlet, entitled *The Importance of the Heredity of Tuberculosis in Comparison with its Propagation by Sputum*, gives a reply to this which goes to prove our position.

It treats of the heredity of tuberculosis, and Dr. Haupt claims and shows that the inheritance of tuberculosis from parents or ancestors cannot possibly be disputed, and that the theory of absolute contagion is not well founded.

One physician had not seen a case of infection after fourteen years' work in a consumption hospital.

Professor Leyden is quoted thus: "Immediately after the discovery of the tubercle bacillus there was a tendency to attach much more importance to contagion than before, but further observation has shown that it does not play so very great a part, and that the majority of cases are due to heredity."

Of six hundred and eighty Italian physicians fifty-nine declared for contagion, one hundred and twenty-four against it, and four hundred and ninety-seven mainly for heredity.

In England seven hundred and ninety-two out of one thousand and seventy-eight declared against contagion.

Here are Dr. Haupt's statistics: Among the fifteen hundred inhabitants of Soden there are one hundred and one who let lodgings. In most of the houses the wives, with sisters or daughters, serve and tend the tuberculous patients who come for treatment. In many houses servant girls from the neighboring villages, hired for the summer, help, making the patients' beds, cleaning their rooms, beating the carpets, removing the sputum. These occupations, it will be seen, are closely connected with the danger of infection. In winter the landlord's family occupy the rooms in which the most seriously affected cases have been. Between 1855 and 1888 forty-eight of the two hundred and thirty-eight members of such families died, ten of them of tuberculosis. In six of these ten cases heredity was demonstrable, and the remaining four were due to colds and external causes. Of the four hundred and fifteen servant girls seventeen died, five of them of tuberculosis, also demonstrably due to other causes than infection. The same proportion prevails among other persons in close contact with consumptive patients, attendants, washerwomen, etc.

We would like to ask, what now becomes of the bacillus origin of tuberculosis? It surely needs more support than has thus far been given it.

In fifteen years' observation we have not seen one case but what showed a hereditary origin.

The reaction of the microbe craze has begun. The advocates of the bacillus and the coccus are now beginning to hedge. It will not be long before the whole idea will be forgotten, and it will then take its place with the numerous other theories of so-called scientific medicine.

G. H. C.

---

Surgery the Opprobrium of Medicine.—At the recent meeting of the International Hahnemannian Association at Watch Hill, Dr. J. B. Bell, of Boston, one of the best surgeons our country has produced, said: "Although I am a surgeon, I say that surgery is the opprobrium of medicine. We should be able to cure all diseases, even those now called surgical, with the indicated homœopathic remedy alone. Surgery should be practiced only in cases of injury. The time will come when surgery will be confined to this field."

Dr. Bell's words find confirmation in a letter of a leading physician of Brussels to a Paris journal. The writer says that the recent progress of surgery has been overrated. That the operative art in itself has not made sensible progress. That since more cleanliness has been practiced the healing of wounds has been attended with greater success than before. The author says: "The real difficulty and the supreme talent of men of the art (of surgery) consist more in preserving than in removing organs, viscera, and tissues, and making tumors disappear by approporiate therapeutical means rather than violently extirpating them. It is thus that a number of so-called cancers of the breast, which are generally removed at once with the knife or with strong caustic, may be easily cured without any painful or cutting operation, in a few months. The statistics of operation carried to extremes are not very reassuring, notwithstanding the incontestable progress accomplished in the hygiene or the salubrity of hospitals, and the excellent care of the patients operated on.

"Every now and then medical journals report that a brilliant operation, performed with all the antiseptic arsenal in vogue, and with a surgical show designed to throw dust in the eyes of the spectators, has been immediately followed by the death of the patient."

In speaking of cancers of the breast, the writer says: "They would become very rare if they were properly treated as soon as puffiness or engorgement manifests itself." The same in the case of goitre. "A cutting operation is far from being without danger."

At the present day, even nearer home, we occasionally see an account in the newspapers of an operation of a serious character having been successfully performed, but the one who gave the item to the reporter fails to mention that the patient died shortly after. Recently such an account of the removal of a tumor from the brain appeared in some Philadelphia newspapers. It is so well described in a portion of the letter quoted above that we shall let the writer of that speak: "That grave operations for medical cases are not always justifiable may be seen by the following observation: About eighteen months ago a well-known surgeon practiced the removal of a tumor of the brain in a patient who had suffered from attacks of unilateral epilepsy for seven or eight years. The tumor was diagnosed to be situated in the convolution of Rolando. The spot for operation having been marked out the surgeon trephined, removing a circle of about three centimetres. He then made a crucial incision into the dura mater, which was healthy, and cutting through the pia mater he incised the brain and removed the tumor, which was about the size of a small apple. During the night preceding the operation there had been thirty-seven epileptic attacks. On the night following it there were only five, and five or six days afterward there were none. Coma and delirium, with which the patient was affected, disappeared toward the tenth day, the complete paralysis of the two limbs, with which he was also affected, disappeared on the fifteenth day, the limbs gradually recovered their movements, and at the end of a month the patient completely recovered consciousness. All this seems very encouraging, but I have incidentally learned that the patient is relapsing into the same condition that he was in before the operation."

What lessons we may draw from this and similar cases! Hahnemann taught the dynamic origin of disease, and experience has shown that, no matter what the manifestations of disease, the only safe and permanent way to health is to follow the law of the similars, by prescribing the remedy demanded by the symptoms. We may rid a patient of the external manifestations of the internal affection by the knife, the cautery or topical applications, but the disease is still at work, and sooner or later it will show in a form even worse than at first, and it will be made incurable by continuing such treatment.

G. H. C.

THE OPPROBRIUM OF OLD-SCHOOL MEDICINE is not only surgery. The results of the treatment of every disease should be a reproach to all who adhere to that lawless drugging method of treating the sick.

Wherever Homœopathy is tried its superior success is shown. Thus, during the epidemic of yellow fever in Jacksonville, Florida, in the winter of 1888–89, old-school mortality was greater than fifteen per cent., while over five hundred cases under Homœopathy showed a mortality of less than three per cent. And still the druggers find feeble imitators who take the name of homœopathist! G. H. C.

---

DR. FRANK KRAFT, of Sylvania, Ohio, editor of *The American Homœopathist*, has been elected Professor of Materia Medica and Lecturer on the *Organon* in the Cleveland Hospital College.

Dr. Kraft has prepared a plan of accurate instruction in the pure principles of Homœopathy. Those who wish to know just what Homœopathy really is, have, at last, an opportunity to hear the doctrine expounded.

At the recent meeting of the International Hahnemannian Association, Dr. Wesselhœft said that students in Harvard and other colleges who desired to learn something about Homœopathy frequently applied to him for information where to go to get this knowledge. He was at a loss to direct them.

Many other homœopathic physicians have been similarly embarrassed.

This difficulty will now be removed by the appointment of Dr. Kraft to the professorship, for it is his fixed intention to give a thorough and complete exposition of the law of cure and of the *Organon*, paragraph by paragraph.

There is a keener demand for genuine homœopathic teaching than the professors of the colleges will believe. For want of it, they stand on a dead level of mediocrity, feebly competing with the successful colleges of the other school.

By cordially upholding Dr. Kraft in his determination to teach Hahnemannian Homœopathy, the Cleveland College will come to the front at once and become distinguished; and it will find itself supported by that small band of Hahnemannians who at present have no college to represent them.

THE HOMŒOPATHIC PHYSICIAN will be happy to lend its aid in support of the new policy. W. M. J.

